# A Case of Myelitis Ending in Recovery*Read before the Suffolk District Medical Society.

**Published:** 1875-06

**Authors:** G. H. Lyman

**Affiliations:** Boston


### A Case of Myelitis Ending in Recovery.*

BY G. H. LYMAN, M. D., OF BOSTON.

The patient, Mrs. E. S. S., aged twenty-five years, was married at nineteen, and has had one child, now five years old; no other conception has occurred. Her parents are healthy. She had typhoid fever six years ago; she has been in good health since, though not as strong as before; her complexion is clear and healthy, her nutrition good.

On the 16th of December she had a severe attack of measels; the attendant cough was very urgent. The disease went through its usual stages without any other complication than the usual severity of the cough, which still remains troublesome. The bowels are constipated. There is slight febrile disturbance, and some pain on pressure over the lumbar vertebræ; there is no muscular twitching; the urine is normal. The patient reports that a week ago (January 1st) she found on awaking in the morning that there was numbness of both feet, with pricking sensations as though the "legs were asleep," though she could stand, and even walk with difficulty. In five or six days the numbness gradually extended to the hips, with manifest increase of loss in muscular power. Hot and cold applications to the spine cause no pain. She is unable to distinguish any difference between a piece of ice and hot water.

January 12th. In attempting to walk across the room the right leg was found to be completely paralyzed, the left nearly so; loss of sensation was nearly complete below the hips, and there was numbness on the right side of the chest.

January 16. Slight relaxation of vesical sphincter.

January 19. Both legs are completely paralyzed, the right more so than the left. If rubbed, forcibly extended, or placed in an uncomfortable position, they become rigid and painful, and spasmodic action is induced, more in the right than the left.

During the past week the spine, below the shoulder blades, has been sufficiently painful to wake her at night. The past two days

*Read before the Suffolk District Medical Society.

the patient has had a decided feeling of stricture about the waist, as though laced too tight. The ominous affection of the vesical sphincter has disappeared. The patient is now unable to stand or walk, and is obliged to be lifted; with much effort she can turn over in bed, and when at rest suffers no pain. Slight headache occasionally, which the patient attributes to medicine. The cough has nearly gone; the appetite is good; the bowels require daily laxative. There is no affection of special senses. The catamenia are regular. Electricity causes imperfect muscular reaction in both extremities, most in the left. The patient was seen to-day by Dr. Ellis in consultation.

When first seen, on January 8th, a laxative pill of quinine, sulphate of iron, colchicum, and aloes was ordered. As the paralysis was not complete, and in the hope that it might be simple congestion of the cord, six leeches were applied to the lumbar region. There being no improvement, but a decided increase of the disease, this was followed in a day or two by ergot and bromide of potassium, a drachm of the former and half a drachm of the latter, three times daily, with good diet and enough morphine and camphor to insure quiet nights.

January 27. In addition to the above, strychnine, one thirtieth of a grain, with fifteen drops of dilute phosphoric acid, was given twice daily alternating with the ergot, one dose of the latter being omitted. She was also given lager beer and still more generous diet.

January 30th. There is some return of sensation, and ability to flex the foot upon the ankle.

Two weeks later (middle of February) the patient was able to rise slowly from her chair without assistance. Ergot and bromide were continued three times daily, the strychnine being omitted. Iodine was applied daily to the lumbar region. Slow but steady improvement continued until March 20th, when the patient was able to walk up and down stairs.

April 1st. The patient has recovered entirely both sensation and motion, with no more weakness than would naturally result from her long confinement. The ergot has been gradually reduced during the past fortnight, and cod-liver oil substituted.

April 3d. Ergot is omitted entirely; the oil is to be continued for a short time.

As recovery from myelitis is rare, it may perhaps be suggested that this was a case of reflex or neurolytic paralysis; but the completeness of the paralysis, the slight pain in the spine caused by pressure on the processes, the stricture about the waist, the pricking sensations, the anæthesia so complete, and the total absence of gastric or urinary derangements, all point to inflammation of the substance of the cord, which fortunately had not progressed so far as to prove intractable to remedies —*Boston Medical and Surgical Journal.*

